# RNAcentral: a comprehensive database of non-coding RNA sequences

**DOI:** 10.1093/nar/gkw1008

**Published:** 2016-10-28

**Authors:** The RNAcentral Consortium, Anton I Petrov, Simon J E Kay, Ioanna Kalvari, Kevin L Howe, Kristian A Gray, Elspeth A Bruford, Paul J Kersey, Guy Cochrane, Robert D Finn, Alex Bateman, Ana Kozomara, Sam Griffiths-Jones, Adam Frankish, Christian W Zwieb, Britney Y Lau, Kelly P Williams, Patricia P Chan, Todd M Lowe, Jamie J Cannone, Robin Gutell, Magdalena A Machnicka, Janusz M Bujnicki, Maki Yoshihama, Naoya Kenmochi, Benli Chai, James R Cole, Maciej Szymanski, Wojciech M Karlowski, Valerie Wood, Eva Huala, Tanya Z Berardini, Yi Zhao, Runsheng Chen, Weimin Zhu, Maria D Paraskevopoulou, Ioannis S Vlachos, Artemis G Hatzigeorgiou, Lina Ma, Zhang Zhang, Joern Puetz, Peter F Stadler, Daniel McDonald, Siddhartha Basu, Petra Fey, Stacia R Engel, J Michael Cherry, Pieter-Jan Volders, Pieter Mestdagh, Jacek Wower, Michael B Clark, Xiu Cheng Quek, Marcel E Dinger

**Affiliations:** 1 1European Molecular Biology Laboratory, European Bioinformatics Institute (EMBL-EBI), Wellcome Trust Genome Campus, Hinxton, Cambridge CB10 1SD, UK; 2 Faculty of Biology, Medicine and Health, University of Manchester, Oxford Road, Manchester M13 9PT, UK; 3 Wellcome Trust Sanger Institute, Wellcome Trust Genome Campus, Hinxton, Cambridgeshire, CB10 1HH, UK; 4 Department of Biomolecular Engineering, University of California Santa Cruz, CA 95064, USA; 5 Department of Biochemistry, University of Texas Health Science Center at San Antonio, 7703 Floyd Curl Drive, San Antonio, TX 78229-3900, USA; 6 Department of Animal Sciences, Auburn University, Auburn, AL 36849, USA; 7 Sandia National Laboratories, Livermore, CA 94551, USA; 8 MRC Functional Genomics Unit, Department of Physiology, Anatomy, and Genetics, University of Oxford, Oxford OX1 3PT, UK; 9 Garvan Institute of Medical Research, Sydney, New South Wales 2010, Australia; 10 Laboratory of Bioinformatics and Protein Engineering, International Institute of Molecular and Cell Biology, Trojdena 4, 02-109 Warsaw, Poland; 11 Frontier Science Research Center, University of Miyazaki, Japan; 12 Institute of Computing Technology, Chinese Academy of Sciences, Beijing 100190, China Institute of Biophysics, Chinese Academy of Sciences, Beijing 100101, China; 13 Data Science, National Center for Protein Science, Beijing, China; 14 Michigan State University, East Lansing, MI 48824-1325, USA; 15 Institute of Molecular Biology and Biotechnology, Faculty of Biology, Adam Mickiewicz University, Umultowska 89, 61-614 Poznan, Poland Department of Computational Biology, Adam Mickiewicz University in Poznan, Poland,; 16 Department of Genetics, Stanford University, Stanford, CA 94305, USA; 17 DIANA-Lab, Department of Electrical & Computer Engineering, University of Thessaly, 382 21 Volos, Greece Hellenic Pasteur Institute, 127 Vasilissis Sofias Avenue, 11521 Athens, Greece; 18 Cambridge Systems Biology Centre & Department of Biochemistry, University of Cambridge, Sanger Building, 80 Tennis Court Road, Cambridge CB2 1GA, UK; 19 Center for Computational Biology and Bioinformatics, The University of Texas at Austin, Austin, TX 78712, USA; 20 The Arabidopsis Information Resource and Phoenix Bioinformatics, 643 Bair Island Rd. Suite 403, Redwood City, CA 94063, USA; 21 "Jacobs University Bremen and Max Planck Institute for Marine Microbiology"; 22 BIG Data Center and CAS Key Laboratory of Genome Sciences and Information, Beijing Institute of Genomics, Chinese Academy of Sciences, Beijing 100101, China; 23 University of Strasbourg, 15 rue R. Descartes, 67084 Strasbourg, France; 24 Bioinformatics Group, Department of Computer Science, and Interdisciplinary Center for Bioinformatics, University of Leipzig, D-04107 Leipzig, Germany; 25 Department of Pediatrics, University of California San Diego, La Jolla, CA, USA; 26 dictyBase, Northwestern University, Chicago, IL, USA; 27 Center for Medical Genetics, Ghent University and Cancer Research Institute Ghent, Ghent University, Ghent, Belgium

## Abstract

RNAcentral is a database of non-coding RNA (ncRNA) sequences that aggregates data from specialised ncRNA resources and provides a single entry point for accessing ncRNA sequences of all ncRNA types from all organisms. Since its launch in 2014, RNAcentral has integrated twelve new resources, taking the total number of collaborating database to 22, and began importing new types of data, such as modified nucleotides from MODOMICS and PDB. We created new species-specific identifiers that refer to unique RNA sequences within a context of single species. The website has been subject to continuous improvements focusing on text and sequence similarity searches as well as genome browsing functionality. All RNAcentral data is provided for free and is available for browsing, bulk downloads, and programmatic access at http://rnacentral.org/.

## INTRODUCTION

Non-coding RNAs (ncRNAs) are a critical component of cellular machinery of all organisms. For example, the ribosomal RNA has been shown to be a ribozyme responsible for peptide bond synthesis (1), and the activity of the eukaryotic spliceosome is mediated by ncRNAs (2). Apart from being the main player in those central processes, ncRNAs provide additional layers of subtle regulation of gene expression. MicroRNAs have been shown to regulate the expression of the majority of mRNAs in animals and plants (3), and the range of regulatory roles of lncRNAs, including by genomic scaffolding and chromatin remodelling and modification (4), is becoming clearer. There is an intense scientific interest in ncRNAs resulting in a large number of ncRNA databases, but until recently searching and comparing them was challenging, and there was no uniform way to access or reference ncRNA sequences. To this end, we developed RNAcentral, a database of ncRNA sequences that serves as a single entry point to the data from a large collection of collaborating ncRNA resources that cover ncRNA sequences of all types and from all organisms. First conceived in 2011 (5), RNAcentral was made public in 2014 (6). This paper gives an update on the status of the database and related activities.

## DATA OVERVIEW

### New Expert Databases

RNAcentral aggregates ncRNA sequence data from an international consortium of RNA resources that we call Expert Databases. In the past two years, 12 additional Expert Databases have been integrated into RNAcentral (see Table 1). Among the newly imported resources were two major ribosomal RNA databases, SILVA (7) and Greengenes (8), which complement rRNAs from ENA and Rfam, as well as a high quality subset of rRNA sequences from RDP (9). Ribosomal RNAs represent the majority of sequences in RNAcentral due to their use in environmental sampling.

**Table 1.** Expert Databases imported into RNAcentral since release 1

Database name	Description	URL
DictyBase	A model organism database for the social amoeba *Dictyostelium discoideum*	http://dictybase.org
Greengenes	A full-length 16S rRNA gene database that provides a curated taxonomy based on de novo tree inference	http://greengenes.secondgenome.com/downloads
LNCipedia	An integrated database of human lncRNAs	http://www.lncipedia.org
MODOMICS	A comprehensive database of RNA modifications	http://modomics.genesilico.pl
NONCODE	An integrated knowledge database dedicated to ncRNAs (excluding tRNAs and rRNAs)	http://www.noncode.org
PDB	A repository of information about the 3D structures of large biological molecules	http://www.wwpdb.org/
PomBase	A comprehensive database for the fission yeast *Schizosaccharomyces pombe*	http://www.pombase.org
SGD	An integrated database for the budding yeast	http://yeastgenome.org
SILVA	A resource for quality checked and aligned ribosomal RNA sequence data	http://www.arb-silva.de/
snoPY	A database of snoRNAs, snoRNA gene loci, and target RNAs as well as snoRNA orthologues	http://snoopy.med.miyazaki-u.ac.jp/
TAIR	A database of genetic and molecular biology data for the model higher plant *Arabidopsis thaliana*	http://www.arabidopsis.org
WormBase	A resource for genomic and genetic data about nematodes with primary emphasis on *Caenorhabditis elegans*	http://www.wormbase.org

Sequences from five Model Organism Databases (DictyBase (10), PomBase (11), SGD (12), TAIR (13) and WormBase (14)) have been imported into RNAcentral. Long non-coding RNA (lncRNA) coverage was extended by the addition of NONCODE (15) and LNCipedia (16) datasets. The inclusion of PDB (17) as an Expert Database helps to map between the worlds of ncRNA sequence and structure. Small nucleolar RNAs (snoRNAs) play an important role in guiding the modification of other ncRNA and mRNAs (18), and snoPY (19) provides to RNAcentral a dataset of snoRNAs found in human, fly, worm, yeast, and thale cress. An up-to-date list of all RNAcentral Expert Databases is available at http://rnacentral.org/expert-databases.

### Database growth

**Figure 1.**
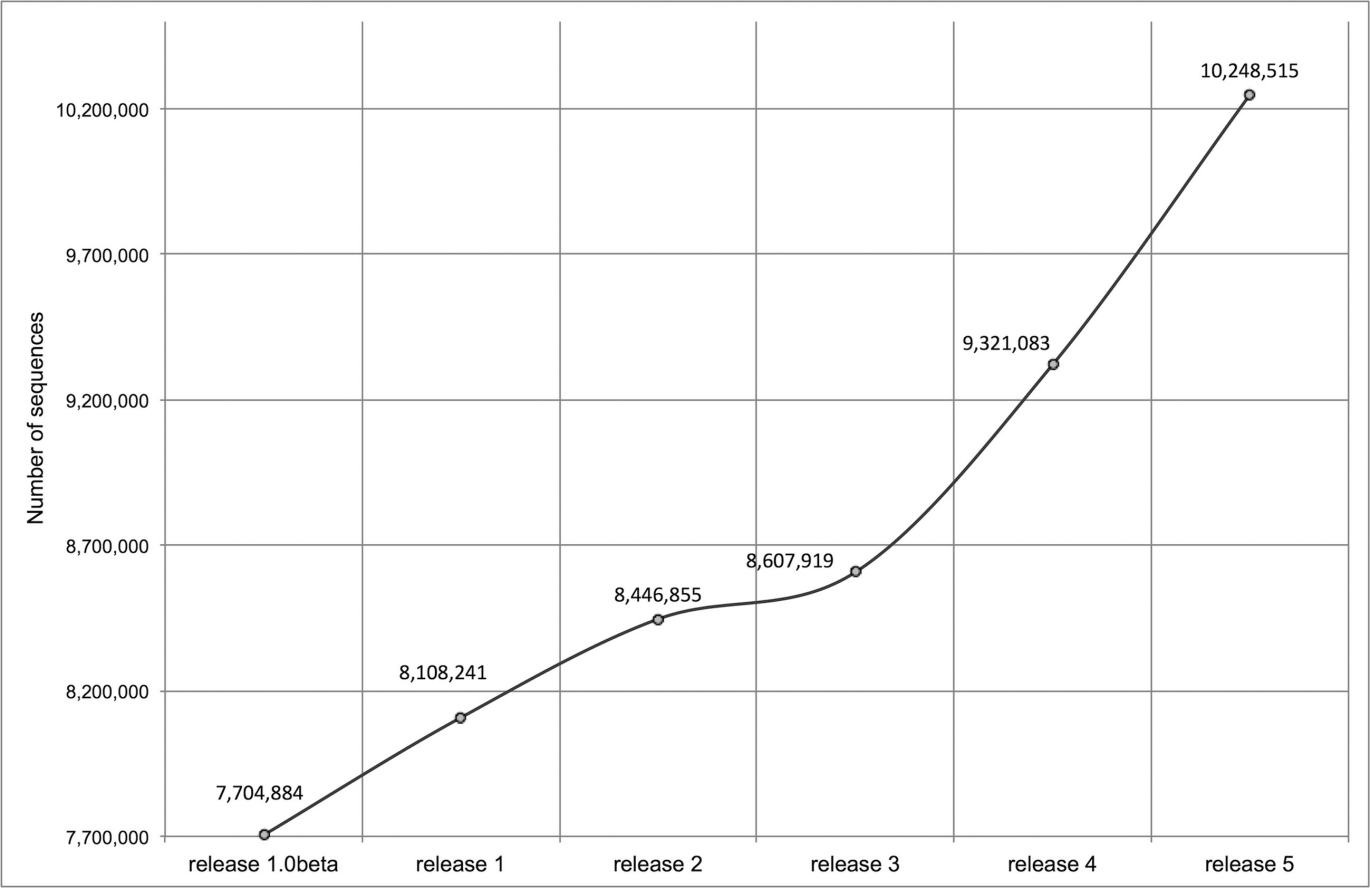
Growth in the number of unique RNA sequences since release 1. An up-to-date version of the chart is available at http://rnacentral.org/about-us.

RNAcentral currently holds 10.2 million distinct ncRNA sequences (an increase of 2.1 million since release 1) with about 28 million cross-references (up 11 million since release 1) to 22 Expert Databases (Figure 1). The sequences come from over 720 000 organisms from all domains of life, with half of all sequences deriving from bacteria and ∼40% from eukaryotes.

### Species-specific identifiers

Since its inception, RNAcentral has provided unique identifiers for each distinct RNA sequence. For example, URS00004C905 is the identifier for the sequence UUUGGUCCCCUUCAACCAGCUG, which is the miRNA miR-133a-3p. The exact same sequence is observed in more than 10 species. These unique and stable identifiers are useful for a number of tasks, such as unambiguously referring to an RNA sequence, reducing redundancy in sequence datasets, and keeping track of cross-references. However, it is essential to be able to refer uniquely to a ncRNA sequence annotation in a particular species. In order to address this issue, we have introduced species-specific RNA sequence identifiers. The new identifiers are composed of a unique RNA sequence identifier and a NCBI taxonomic identifier separated by underscore. For example, URS00004C9052_9606 is the human copy of the miRNA hsa-miR-133a-3p (*9606* being the taxonomic identifier for *Homo sapiens*) and URS00004C9052_10090 corresponds to the mmu-miR-133a-3p sequence from mouse. RNAcentral text searches now return species-specific entries by default.

One of the use cases for the species-specific RNA sequence identifiers is curation of literature references to assign ontology terms to an RNA sequence from a specific organism, for example to annotate the molecule's function. The biocuration community has already begun using RNAcentral identifiers for assigning Gene Ontology terms to human miRNAs (20). We are investigating assigning identifiers that differentiate between multiple occurrences of the same sequence in a genome.

### Modified nucleotides

**Figure 2.**
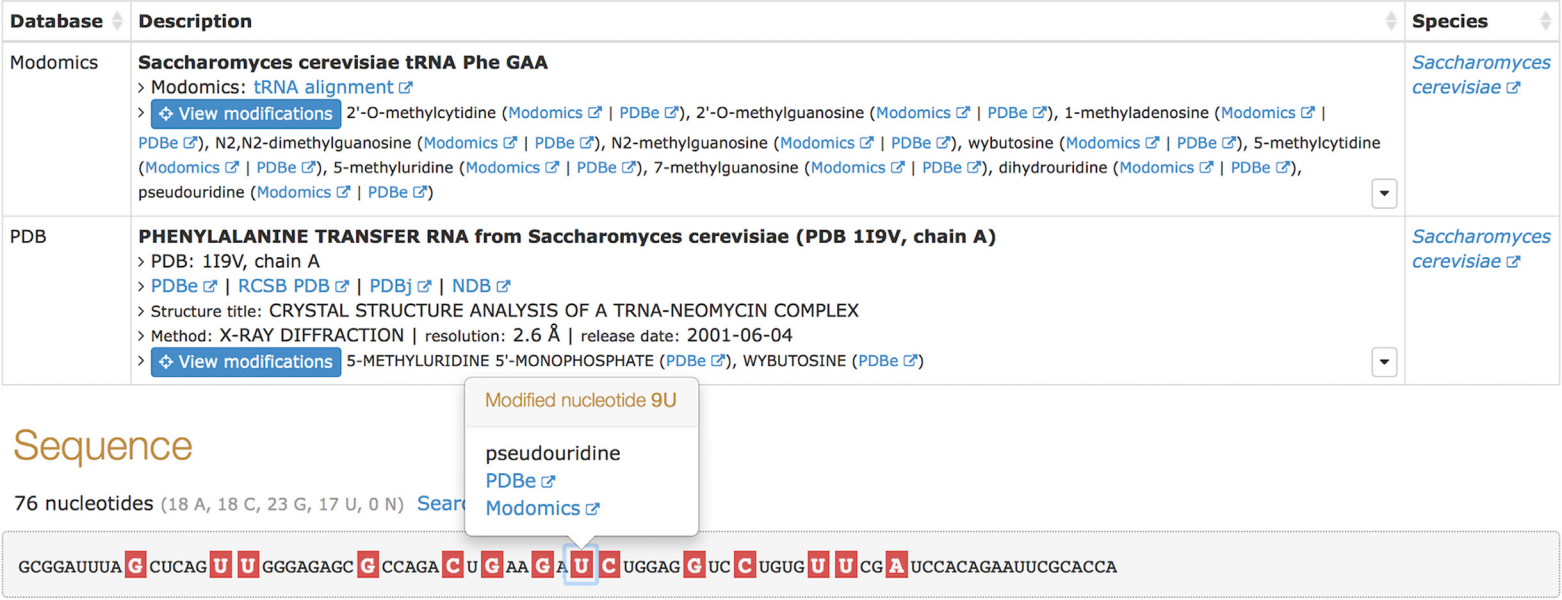
Web interface displaying modified nucleotides for a *Saccharomyces cerevisiae* tRNA(Phe) sequence (RNAcentral entry URS000011107D_4932).

Modified nucleotide residues play important roles in functions of many ncRNAs. For example, modifications of ribosomal RNAs are essential for the assembly and stability of ribosomes (21), and tRNA modifications can influence protein gene expression (22). In order to begin capturing information about modified residues in RNA molecules and enable comparison of different datasets, we imported modified rRNA and tRNA sequences from MODOMICS (23), a database of RNA modification pathways, as well as all ncRNA modifications from Protein Data Bank. So far RNAcentral contains over 170 different chemical modifications found at over 8000 positions in about 600 unique sequences. Figure 2 shows a web interface that provides a unified view of the modifications from different databases. For each modified residue, cross-references to the MODOMICS and PDB databases are provided to enable easy access to more detailed information about each modification. In future releases we will continue importing information about modified nucleotides from MODOMICS and other resources as more data become available thanks to new developments in sequencing technology (24,25).

## WEBSITE UPDATES

The RNAcentral website has been subject to continuous improvement based on user feedback and several interactive workshops held during annual RNAcentral consortium meetings. The homepage was redesigned to reflect the three main ways to access the data: **text search, sequence similarity search** and **genome browser**, each of which will be discussed further below.

### Text search

RNAcentral text search provides a flexible way for exploring RNAcentral data using a faceted interface powered by EBI search (26). In the past two years, the search functionality was improved both in terms of user interface and searchable data. For example, publication metadata (such as paper titles, PubMed identifiers or author names) associated with ncRNA sequences can now be searched, which makes it possible to look up ncRNA sequences submitted to sequence archives when new papers are published. For example, a recent paper describes TRM10 (27), a mRNA-derived small RNA that acts as ribosome inhibitor. By searching RNAcentral with PubMed identifier *24685157* the sequence is easily found (see entry URS00007E15D1) and can be used for further analysis, such as sequence similarity search. Moreover, it is possible to compare sequences reported in different papers. For example, one can identify mitochondrial rRNA sequences shared by a Danish (28) and an Iranian (29) population by searching in RNAcentral with both publication titles. These search results can be exported in multiple formats, thereby facilitating more detailed investigation by the user.

### Sequence similarity search

RNAcentral sequence search is the first online tool that enables sequence similarity searches against a comprehensive set of ncRNAs. The service is powered by the *nhmmer* software, which has a comparable speed to BLAST but is more sensitive (30). The web interface supports searching using an RNA or DNA sequence as a query and displays pairwise sequence alignments for each match. The results can be sorted by E-value, sequence identity and other criteria. If an exact match for a query sequence already exists in the database, its RNAcentral identifier is retrieved using the RNAcentral API without having to wait for the full search results to become available.

### Genome browser

**Figure 3.**
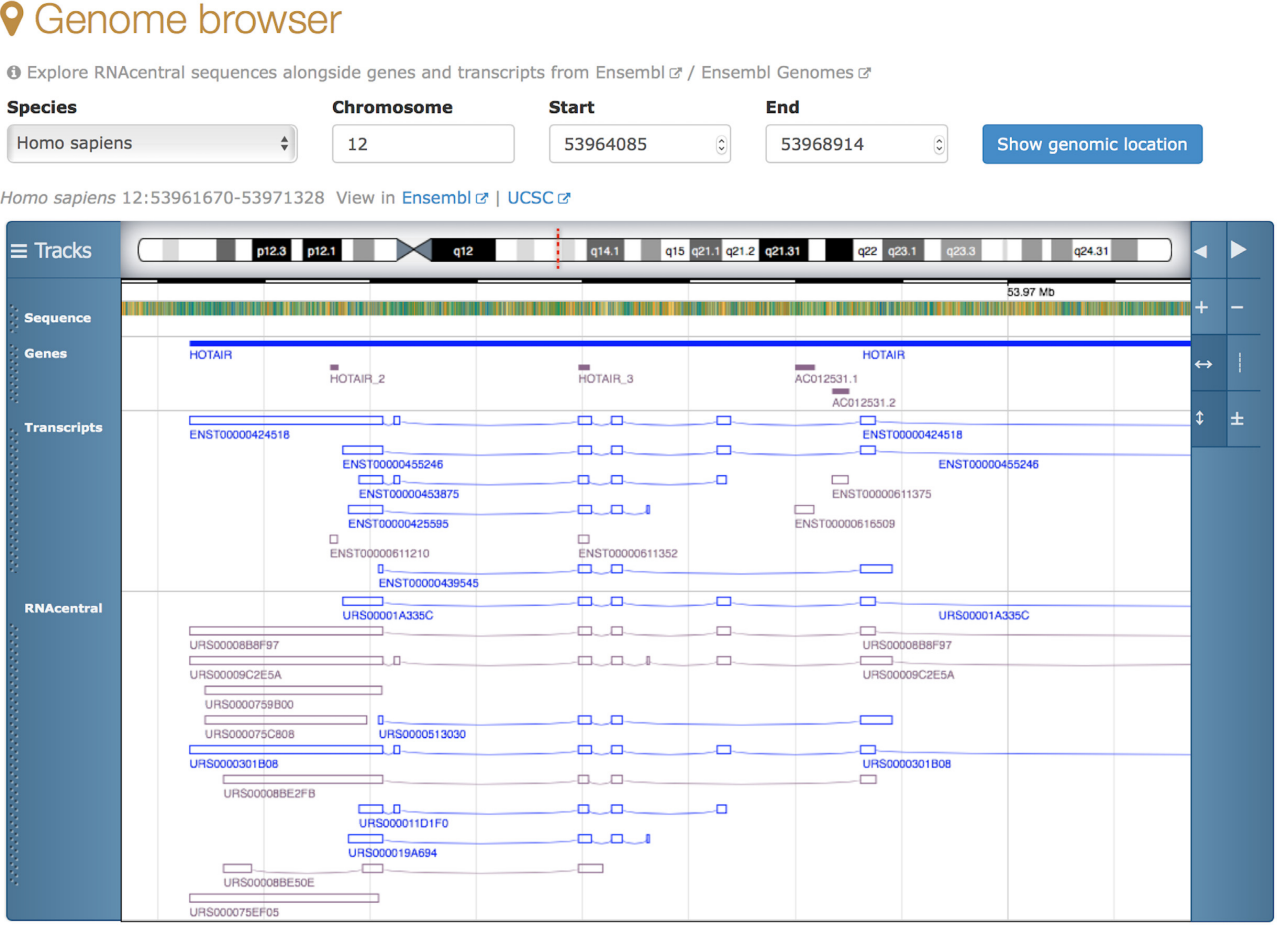
RNAcentral genome browser showing HOTAIR lncRNA in human chromosome 12.

Viewing sequences in their genomic context can provide important biological insights. For example, one can visualise snoRNAs found in introns of lncRNA GAS5 (31) using a built-in genome browser (see RNAcentral entry URS00008B3C85). In a recent update, we extended this functionality to enable browsing RNAcentral starting with a genomic location. The embedded genome browser, powered by Genoverse (http://genoverse.org), currently supports 13 key species, including human, mouse, fly, worm, and yeast (Figure 3). RNAcentral sequences are displayed alongside genes and transcripts from Ensembl (32) and Ensembl Genomes (33) with links to fully featured genome browsers, such as UCSC (34) and Ensembl. The genomic data are also available via a programmatic interface and downloadable files in BED/GFF formats.

## RNACENTRAL USE CASES

Citations to RNAcentral are beginning to appear in the scientific literature, and currently fall into three main categories of use: (i) RNAcentral is used as a comprehensive source of ncRNA annotations and a reference for identification of novel ncRNAs in species like rainbow trout or cow (35,36). (ii) RNAcentral identifiers are used for literature curation, for example, human miRNAs are annotated with GO terms using RNAcentral species-specific identifiers to refer to RNA sequences (20). (iii) The RNAcentral API is used for sequence or identifier retrieval (37–39). For example, given an RNAcentral sequence identifier the Forna tool can predict and visualise its secondary structure. Over the past two years, the RNAcentral website has been accessed by over 33 000 unique visitors from 156 countries who performed over 100 000 text and 12 000 sequence similarity searches.

## TRAINING AND OUTREACH

We continuously engage in outreach activities and provide user support by email and on GitHub. We have delivered over 20 presentations to date at scientific conferences and research institutes worldwide and organised a training event at the Wellcome Genome Campus. We also developed an online training course and recorded a live webinar (available on YouTube). All training materials can be accessed at http://rnacentral.org/training. We are open to suggestions from our user community by email, on GitHub and on Twitter. The contact information can be found at http://rnacentral.org/contact.

## FUTURE DIRECTIONS

The main goal of RNAcentral is to provide a comprehensive set of ncRNA sequences, so integrating new Expert Databases and importing more data will remain a priority. For example, our goal is to integrate the remaining Model Organism Databases, such as FlyBase (40) and RGD (41), in order to provide uniform access to high-quality ncRNA sequence and annotations from key species. More than 20 participating ncRNA resources still need to be integrated and new Expert Databases are continually joining the consortium. We welcome relevant databases to contact us about membership.

In the second phase of development, we will begin to integrate new types of annotations that can provide insight into the functions of ncRNA sequences found in RNAcentral. We will work on importing secondary structure information from Comparative RNA Website (42), GtRNAdb (43) and Rfam (44) databases. Work is underway on integrating miRNA-mRNA interactions from TarBase (45) and miRNA–lncRNA interactions from LncBase (46) into RNAcentral. We will enrich existing annotations by importing ontology terms from external resources and assigning ontology terms automatically where possible. We also plan to use sequence alignment-based mapping to connect more RNAcentral sequences to reference genomes. RNAcentral is a young and fast-growing resource, but it has already proved useful for many applications, and its utility will be increased as more data are integrated and the associated services mature.
